# Making myelin basic protein -from mRNA transport to localized translation

**DOI:** 10.3389/fncel.2013.00169

**Published:** 2013-09-27

**Authors:** Christina Müller, Nina M. Bauer, Isabelle Schäfer, Robin White

**Affiliations:** Institute of Physiology and Pathophysiology, University Medical Center of the Johannes Gutenberg UniversityMainz, Germany

**Keywords:** myelin basic protein, hnRNP A2, mRNA localization, oligodendrocyte, Fyn, RNA granule, translational regulation, review

## Abstract

In the central nervous system (CNS) of most vertebrates, oligodendrocytes enwrap neuronal axons with extensions of their plasma membrane to form the myelin sheath. Several proteins are characteristically found in myelin of which myelin basic protein (MBP) is the second most abundant one after proteolipid protein. The lack of functional MBP in rodents results in a severe hypomyelinated phenotype in the CNS demonstrating its importance for myelin synthesis. *Mbp *mRNA is transported from the nucleus to the plasma membrane and is translated locally at the axon–glial contact site. Axonal properties such as diameter or electrical activity influence the degree of myelination. As oligodendrocytes can myelinate many axonal segments with varying properties, localized MBP translation represents an important part of a rapid and axon-tailored synthesis machinery. MBP’s ability to compact cellular membranes may be problematic for the integrity of intracellular membranous organelles and can also explain why MBP is transported in oligodendrocytes in the form of an mRNA rather than as a protein. Here we review the recent findings regarding intracellular transport and signaling mechanisms leading to localized translation of *Mbp *mRNA in oligodendrocytes. More detailed insights into the MBP synthesis pathway are important for a better understanding of the myelination process and may foster the development of remyelination therapies for demyelinating diseases.

## MYELIN BASIC PROTEIN

In the central nervous system (CNS) oligodendrocytes produce and maintain myelin. The insulating multilamellar myelin sheath improves neuronal communication by increasing impulse propagation velocity in neuronal axons while limiting energy requirements. It is also becoming more and more apparent that oligodendrocytes provide trophic support for neuronal axons which is essential for axonal survival and integrity (Funfschilling et al., 2012; Lee et al., 2012). A lack or disruption of intact myelin is associated with several neurological disorders including multiple sclerosis and inherited leukodystrophies.

One of the major components of CNS myelin is myelin basic protein (MBP; Jahn et al., 2009), which has been referred to as the “executive molecule of myelin” (Boggs, 2006). Shiverer (*shi*) mice as well as long evans shaker (*les*) rats both lack functional MBP and are characterized by severe hypomyelination in the CNS, shivering symptoms, and premature death (Readhead and Hood, 1990; Kwiecien et al., 1998). Interestingly, compact myelin can be formed in the peripheral nervous system of both mutant rodents which seems to depend on a compensatory function of the P0 protein which is not expressed in the CNS (Martini et al., 1995). The deficiency of other myelin proteins such as proteolipid protein (PLP) and 2′,3′-cyclic nucleotide 3′-phosphodiesterase (CNP) seems to cause secondary neuronal effects rather than affecting CNS myelination as such (Klugmann et al., 1997; Griffiths et al., 1998; Lappe-Siefke et al., 2003).

The multifunctional classic MBP proteins arise from a gene complex called *Golli* (genes of oligodendrocyte lineage) which also gives rise to the Golli (-MBP) family of proteins (Campagnoni et al., 1993). The *Golli* gene has three different transcriptional start sites allowing the expression of the two distinct subfamilies of proteins, which are temporally and locally regulated. Whereas the presence of classic MBP proteins is mainly restricted to myelinating cells, Golli (-MBP) proteins have been described in other neural and non-neural cells (Fulton et al., 2010). The different MBP isoforms in the mouse (14, 17.22, 17.24, 18.5, 20.2, and 21.5 kDa) mainly stem from transcription start site 3 and are formed by differential splicing (Harauz and Boggs, 2013). All classic MBP isoforms are encoded by exons I, III, IV, and VII, while exon II, V, and VI are only found in specific splice variants.

Interestingly, different isoforms are developmentally regulated and have different cellular distributions. Exon II-containing isoforms (17.22, 20.2, and 21.5 kDa) are expressed at high levels in early development, are spread throughout the cytoplasm and also accumulate in the nucleus (Allinquant et al., 1991; Smith et al., 2013). Nuclear 21.5 kDa MBP appears to influence the proliferation of immortalized N19 oligodendroglial cells and stimulates morphological changes in co-cultured neuronal N2a cells (Smith et al., 2013). Exon II-containing MBPs have also been found in compact myelin but appear to be enriched in the radial component of myelin (Karthigasan et al., 1996).

MBP Isoforms lacking exon II are located at the plasma membrane (Allinquant et al., 1991). Due to its positive charge MBP associates with the negatively charged oligodendroglial phospholipids and has traditionally been proposed to function primarily in the compaction of myelin membranes. It was shown in mice and zebrafish that phosphatidylinositol 4,5-bisphosphate (PIP2) recruits MBP to the plasma membrane and that this interaction can be counteracted by elevated calcium levels (Nawaz et al., 2009, 2013). MBPs function in myelin compaction by membrane association is obviously very important, but additional functions have been assigned to this molecule and may explain the drastic phenotypes observed in its absence.

It was recently shown that MBP protein is involved in regulating the protein to lipid ratio of myelin membranes by acting as a molecular sieve and by inhibiting the diffusion of membrane proteins with large cytosolic domains into myelin membrane sheets (Aggarwal et al., 2011). In these developing membrane sheets, MBP seems to oligomerize into a cohesive protein meshwork which drives other myelin proteins such as myelin-associated glycoprotein (MAG) or CNP out of the sheet to form a lipid rich insulating myelin membrane with only few remaining proteins, largely PLP and MBP (Aggarwal et al., 2013).

It has also been demonstrated that MBP interacts with cytoskeletal proteins and influences their bundling and polymerization (Dyer et al., 1994; Hill and Harauz, 2005; Hill et al., 2005).

In addition to these structural tasks, MBP has been connected to signaling pathways. MBP has the ability to bind signaling molecules such as Fyn kinase which is important for morphological differentiation and myelination (Kramer-Albers and White, 2011; Smith et al., 2012). Moreover, MBP binding to the plasma membrane modulates voltage-operated Ca^2^^+^ channels (VOCCs) and thereby affects Ca^2^^+^ responses in the cell (Smith et al., 2011).

The distinct functions as well as their regulation by post-translational modifications are reviewed in detail elsewhere (Boggs, 2006; Harauz et al., 2009; Harauz and Boggs, 2013) and emphasize that MBP is an essential protein for many aspects of oligodendrocyte homeostasis and myelin formation. Here we review the synthesis of MBP and focus on the posttranscriptional events including mRNA transport and localized translation.

## *Mbp* mRNA IS LOCALIZED IN RNA TRANSPORT GRANULES

As *Mbp* mRNA and ribosomes were found to be present in biochemically purified myelin fractions thirty years ago, it was postulated that *Mbp* mRNA is transported to the myelin compartment where translation occurs locally (Colman et al., 1982). This study also demonstrated the efficiency of this localization system. It was shown that newly synthesized MBP protein can be detected in myelin fractions as early as 2 min after translation in the actively myelinating brainstem of young rats. Following this, microinjection experiments with labeled *Mbp* mRNA revealed the formation of RNA transport granules which are moved on microtubules throughout the cytoplasm to the distal parts of oligodendrocyte processes (Ainger et al., 1993; Carson et al., 1997).

Cytoplasmic mRNA localization has been described for many mRNAs and cell types (Shahbabian and Chartrand, 2012). Commonly, different RNA binding proteins referred to as *trans*-acting factors bind to specific nucleotide sequences in the mRNA’s 3′untranslated region (UTR) termed *cis*-acting factors or elements. mRNA localization can expand the control of cellular gene expression in a spatio-temporal manner by inhibiting mRNA translation until a specific location is reached at a defined time point.

The formation of RNA granules including a specific selection of the transported cargo needs to be tightly controlled. Members of the QKI family of proteins have been reported to influence nucleo-cytoplasmic transport as well as stabilization of *Mbp* mRNA (Li et al., 2000; Larocque et al., 2002; Bockbrader and Feng, 2008; Wang et al., 2010).

Cytoplasmic transport of *Mbp* mRNA largely depends on an RNA binding protein termed heterogeneous nuclear ribonucleoprotein (hnRNP) A2 which binds to a specific sequence in the 3′UTR (Hoek et al., 1998). This sequence was initially termed the RNA transport signal (RTS) consisting of 21 nucleotides (Ainger et al., 1997) and it was subsequently shown that hnRNP A2 binding requires only 11 nucleotides (GCCAAGGAGCC) which are referred to as the A2 response element (A2RE; Munro et al., 1999). This *cis*-acting A2RE has been identified in several other mRNAs including glial myelin-associated oligodendrocytic basic protein (*Mobp*), *Tau*, carbonic anhydrase II (*CaII*), amyloid precursor protein (*App*), as well as neuronal calcium/calmodulin-dependent protein kinase IIα (*CamKIIα*), *Neurogranin*, and activity-regulated cytoskeleton-associated protein (*Arc*; Barbarese et al., 1999; Gao et al., 2008).

Alternative splicing of pre-mRNA encoded by the *HnRNP A2/B1/B0* gene results in the synthesis of the four isoforms hnRNP B1, A2, B1b, and A2b (Hatfield et al., 2002). In most of the studies dealing with hnRNP A2 and *Mbp* mRNA, antibodies were used which do not distinguish between the four isoforms in immunocytochemical experiments. Hence the drawn conclusions focused on the most abundant hnRNP A2 protein. It was recently suggested that hnRNP A2b is the predominant isoform in the cytoplasm of neural cells and that *Mbp* mRNA granule formation seems to depend on hnRNP A2b (Han et al., 2010). It may be the case that the described cytoplasmic functions of hnRNP A2 in the context of *Mbp* mRNA localization which we review here should be (mainly) attributed to hnRNP A2b and be taken into account when we refer to hnRNP A2.

The A2RE is encoded as part of exon VII and is hence present in every *Mbp* splice variant. Therefore, all of the *Mbp *mRNAs can potentially interact with hnRNP A2 and RNA granules. Interestingly, it was proposed that similar to the protein, also exon II-containing mRNAs are differentially localized in oligodendrocytes. It seems that exon II-containing *Mbp* mRNAs are located in the cell body whereas the exon II-lacking mRNAs are transported into the distal cellular processes (de Vries et al., 1997). It remains unclear how the exon II sequence influences the subcellular distribution of *Mbp* mRNAs encoding these 17.22, 20.2, and 21.5 kDa isoforms.

In addition to the A2RE, *Mbp* mRNA contains an additional *cis*-acting sequence in its 3′UTR termed the RNA localization region (RLR) (Ainger et al., 1997) or RNA localization signal (RLS; Barbarese et al., 1999). It has been proposed that the secondary structure of this sequence is required for specific localization of *Mbp* mRNA into the myelin compartment.

Recently, additional RNA binding proteins were identified in *Mbp* mRNA granules (Raju et al., 2008; Laursen et al., 2011; White et al., 2012). HnRNP K as well as hnRNP F are both associated with *Mbp* mRNA and hnRNP A2 and influence the synthesis of MBP protein (Laursen et al., 2011; White et al., 2012). Coimmunoprecipitation of hnRNP F with hnRNP A2 is RNAse resistant while the copurification of hnRNP A2 with hnRNP K seems to depend on RNA. Thus hnRNP F is possibly recruited to the RNA granule by hnRNP A2 while hnRNP K binds to *Mbp* mRNA directly to a yet undefined region. The knock down of hnRNP K appears to abolish the transport of *Mbp* mRNA from process branch points to the most distal parts of oligodendrocyte processes and suggests a role of hnRNP K during this part of the transport path (Laursen et al., 2011). Earlier studies had already observed oligodendroglial hnRNP K in granular structures in the more proximal and not the distal parts of the processes (Kosturko et al., 2005), but it seems that the cellular distribution of hnRNP K depends on the differentiation status of the oligodendrocyte (Laursen et al., 2011).

In addition to hnRNP K and hnRNP F, the A/B type hnRNP CBF-A was identified in *Mbp* mRNA granules. CBF-A binds to the RTS of *Mbp* mRNA, coimmunoprecipitates with hnRNP A2, A3 as well as U and knock down of CBF-A in the immortalized oligodendrocyte precursor cell (OPC) line Oli-*neu* inhibits the transport of *Mbp* mRNA to the processes (Raju et al., 2008). It appears that hnRNP K and CBF-A are important for different parts of the transport path from the cytosol to the most distal regions of the processes, but this needs to be addressed in more detail in the future.

HnRNP F is tyrosine-phosphorylated by Fyn kinase (White et al., 2012) and hnRNP K appears to become tyrosine-phosphorylated in oligodendrocytes cultured on laminin for 4 days (Laursen et al., 2011). Although it has been shown that laminin stimulates oligodendroglial Fyn activity and that Fyn interacts with hnRNP K in the CNS (Kai et al., 1997), it remains to be shown if hnRNP K is a target of Fyn. The influence of Fyn activation on translation of MBP is discussed below.

It was postulated that *Mbp* mRNA is transported on microtubules to the myelin compartment and that this transport requires kinesin as the translocation of microinjected *Mbp* mRNA to the myelin sheets of cultured oligodendrocytes is perturbed by drugs affecting microtubule dynamics or by kinesin antisense RNA treatment (Carson et al., 1997). Furthermore, oligodendrocytes derived from the hypomyelinated *taiep* rat mutant have abnormally accumulated microtubules and show restricted transport of *Mbp* mRNA granules (Song et al., 2003). As shown in zebrafish, the kinesin motorprotein Kif1b is required for the transport of *Mbp* mRNA toward the processes of myelinating oligodendrocytes and Kif1b mutants show ectopic localization of MBP protein as well as misplaced myelin like membranes (Lyons et al., 2009). The microtubule associated protein tumor overexpressed gene (TOG) colocalizes with *Mbp* mRNA and hnRNP A2 in granules and might be involved in the regulation of kinesin activity, thereby directing granule transport toward the plus end of the microtubule (Kosturko et al., 2005).

## TRANSLATIONAL REPRESSION DURING *Mbp* mRNA TRANSPORT

It has been proposed that RNA granules contain all necessary molecules for the translation of mRNA and indeed a number of relevant molecules have been identified in *Mbp* mRNA granules. These include arginyl-tRNA synthetase (ATS), elongation factor 1a (EF1a), and ribosomal RNA (Barbarese et al., 1995). If mRNA granules are more or less ready to translate the transported mRNAs, then premature or ectopic translation must be avoided until certain signals set off the protein synthesis machinery. Regarding *Mbp* mRNA, hnRNP E1 and the small non-coding RNA 715 (sncRNA715) have been directly connected to translational inhibition during transport (Kosturko et al., 2006; Bauer et al., 2012), while the function of other granule molecules such as hnRNP F, TOG, and hnRNP K in respect to translational control may have a more indirect effect on the MBP synthesis path (Francone et al., 2007; Laursen et al., 2011; White et al., 2012).

HnRNP E1 partially colocalizes with hnRNP A2 and microinjected A2RE-containing mRNA in oligodendrocytes (Kosturko et al., 2006). Furthermore hnRNP E1 inhibits the translation of an A2RE-containing *Green fluorescent protein* reporter mRNA in microinjected B104 neuroblastoma cells and in a rabbit reticulocyte lysate-based *in vitro* assay (Kosturko et al., 2006). Additional experiments lead to the conclusion that hnRNP E1 is recruited to RNA granules by hnRNP A2 and inhibits the translation of A2RE-containing mRNAs during transport (Kosturko et al., 2006).

In the light of the emerging importance of post-transcriptional gene regulation by small RNA molecules such as microRNAs or endogenous siRNAs, translational inhibition of localized mRNAs by these types of molecules appears to be a suitable mechanism and has been reported in neurons (McNeill and Van Vactor, 2012). Recently, sncRNA715 was revealed to inhibit the synthesis of endogenous MBP in primary oligodendrocytes via binding to a specific recognition site in the 3′UTR of *Mbp* mRNA (Bauer et al., 2012). SncRNA715 copurifies with hnRNP A2 and *Mbp* mRNA biochemically and is located in granular structures in the cytoplasm and processes of cultured oligodendrocytes. Interestingly, the analysis of chronic multiple sclerosis lesions containing OPCs and *Mbp* mRNA but lacking MBP protein revealed abnormally high levels of sncRNA715 compared to normal appearing white matter (NAWM; Bauer et al., 2012). These data not only allude to an evolutionary conserved mechanism of MBP translational regulation, but may also contribute to a better understanding of why OPCs fail to differentiate appropriately to remyelinate axons in late stages of MS.

While yet unclear for hnRNP E1, sncRNA715 seems to regulate all MBP isoforms as the binding sequence is present in all of the *Mbp* mRNA 3′UTRs and Western blot analysis revealed a translational repression of every isoform (Bauer et al., 2012). So far it is unknown if hnRNP E1 and sncRNA715 synergistically influence MBP translation in oligodendrocytes or if they act at different time points or cellular locations.

Conceptually, one could distinguish two aspects of translational repression of *Mbp* mRNA. As *Mbp* mRNA can be detected in OPCs which do not yet synthesize MBP protein, MBP translation may generally be repressed in OPCs until a certain degree of differentiation has been reached at which defined (axonal) signals initiate MBP protein synthesis. In myelinating oligodendrocytes MBP protein synthesis may be repressed during intracellular transport to prevent ectopic localization of MBP which may have deleterious consequences by compacting intracellular membranes (Staugaitis et al., 1990). The latter also appears to be very important for a specific synthesis machinery capable of producing defined amounts of myelin in response to axonal determinants such as axon diameter or activity.

**FIGURE 1:**
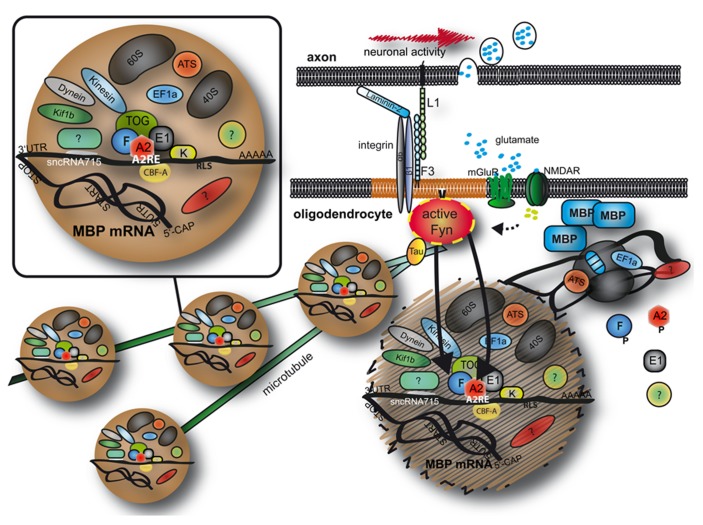
**Transport and localized translation of MBP.** *Mbp* mRNA is transported on microtubules toward the oligodendroglial plasma membrane in RNA granules containing RNA binding proteins, motor proteins and parts of the (if not the entire) protein synthesis machinery. At the plasma membrane, Fyn kinase is a crucial signaling molecule and converts axonal signals into localized translation of MBP. See text for details. MBP, myelin basic protein; A2, F, E1, K, heterogeneous nuclear ribonucleoproteins A2, F, E1, and K; CBF-A, CArG-box binding factor A; TOG, tumor overexpressed gene; mGluR, metabolic glutamate receptor; NMDAR, NMDA receptor; EF1a, elongation factor 1a; ATS, arginyl-tRNA synthetase; 60S/40S, large/small ribosomal subunit; 5′CAP, 5′ 7-methylguanylate CAP; UTR, untranslated region; AAAAA, Poly A tail.

A number of molecules participate in the transport of *Mbp* mRNA in oligodendrocytes and are proposed to function in RNA granule formation, transport and translational inhibition (summarized in **Table 1** and **Figure 1**). RNA deep sequencing and advanced proteomics will most likely identify additional regulatory molecules in the future.

**Table 1:** Molecules associated with MBP mRNA during cytoplasmic localization as mentioned in the text.

Molecule	Binding region	Reference
hnRNP A2	A2RE within RTS in 3′UTR	Hoek et al. (1998)
hnRNP K	Undefined	Laursen et al. (2011)
hnRNP F	Presumably via hnRNP A2	White et al. (2012)
hnRNP CBF-A	RTS in 3′UTR	Raju et al. (2008)
TOG	Presumably via hnRNP A2	Kosturko et al. (2005)
sncRNA 715	Specific recognition site in 3′UTR	Bauer et al. (2012)
hnRNP E1	Presumably via hnRNP A2	Kosturko et al. (2006)

## TRANSLATIONAL DE-REPRESSION AND LOCAL MBP SYNTHESIS

Translationally inhibited mRNAs in RNA granules have been shown to be de-repressed in order to initiate protein synthesis at a specific time point or localization in cells (Ostareck-Lederer et al., 2002; Huttelmaier et al., 2005).

As mentioned above oligodendrocytes must regulate the amount of myelin that is produced at specific axonal segments and as these amounts may vary, it seems to be the case that at least parts of the myelin synthesis machinery is decentralized to respond to axonal requirements locally. Src-family non-receptor tyrosine kinases have been implicated with the initiation of localized translation of transported mRNAs by phosphorylation of *trans*-acting factors (Ostareck-Lederer et al., 2002; Huttelmaier et al., 2005).

In oligodendrocytes Fyn kinase is the predominant Src-family kinase and is an important regulator of myelination (Kramer-Albers and White, 2011). In a screen for Fyn substrates in oligodendroglial cells, hnRNP A2 was identified and an axonal–glial signaling pathway was suggested controlling the activation of Fyn in order to trigger MBP translation at the axon–glial contact site (White et al., 2008). It was shown that binding of the axonal cell adhesion molecule L1 to oligodendroglial F3/Contactin activates Fyn kinase which phosphorylates hnRNP A2 and promotes translation of A2RE-containing mRNAs (White et al., 2008).

It was later contributed that F3/Contactin forms a complex with α6β1 integrins and that L1 binding enhances myelination in a co-culture system which can be blocked by the addition of antibodies directed against β1 integrins (Laursen et al., 2009), suggesting a coordinated regulation of Fyn activation and myelination by the extracellular matrix and the axonal surface.

The stimulation of α6β1 integrins by laminin binding increases Fyn activity (Laursen et al., 2009) and the presence of a constitutively active β1 integrin mutant promotes MBP translation (Laursen et al., 2011). As described above the granule protein hnRNP F is phosphorylated by Fyn. Active Fyn releases hnRNP F from the granule and reduces the amount of *Mbp* mRNA bound to hnRNP F (White et al., 2012). As hnRNP A2 and hnRNP E1 also appear to be released from the granule in these conditions (White et al., 2008) it seems that Fyn activation results in a breakdown of the granule by phosphorylation of granule proteins, releasing *Mbp* mRNA from its inhibitors at the axon–glial contact site and allowing localized MBP protein synthesis to occur. It may be the case that sncRNA715 is also separated from *Mbp* mRNA in response to Fyn activity by a yet unknown mechanism.

Interestingly, it was recently shown that electrical stimulation increases the axonal surface expression of L1 as well as Fyn activation, local MBP synthesis and myelination all of which appears to be regulated by axonal vesicular glutamate release (Wake et al., 2011).

All of the above mentioned studies emphasize the central role of Fyn kinase in translational regulation of MBP. This has strong implications for the myelination process, as demonstrated *in vivo*, as mice are hypomyelinated in the CNS in the absence of Fyn or in the presence of mutated inactive Fyn (Sperber et al., 2001). Moreover, MBP levels are reduced in Fyn knockout mice and Fyn phosphorylates QKI proteins modulating binding and stabilization of *Mbp* mRNAs (Zhang et al., 2003; Lu et al., 2005). In an elegant recent *in vivo* myelination study in zebrafish it was shown that Fyn regulates the number of myelin sheaths per oligodendrocyte (Czopka et al., 2013).

Intriguingly, active Fyn binds to α-Tubulin as well as the microtubule associated protein Tau in oligodendrocytes and it was proposed that activated Fyn recruits the cytoskeleton toward the axon–glial contact site (Klein et al., 2002). As mentioned above, *Mbp* mRNA granules are transported on microtubules toward the periphery of the cell, so that activation of Fyn could recruit *Mbp* mRNA to the axon–glial contact site to initiate the myelination process by synthesizing MBP. However, it is likely that also Fyn-independent pathways regulate the translation of *Mbp* mRNA. Interestingly, a function of TOG in the synthesis of MBP has been suggested which does not seem to be dependent on granule transport (Francone et al., 2007). Nevertheless, a lot of data provide evidence for an intercellular signaling pathway originating from active neuronal axons which recruits *Mbp* mRNA and induces localized MBP synthesis and myelination.

## CONCLUSION

The overall synthesis of MBP is a complex and highly regulated mechanism and we have only discussed elements of it here. As summarized in **Figure 1**, *Mbp* mRNA is transported in large ribonucleoprotein complexes on microtubules toward the oligodendroglial plasma membrane. A number of proteins and RNAs form these granules and MBP translation is repressed during transport. It seems that axon–glial signaling events recruit the granules and stimulate localized translation of MBP allowing axon-tailored myelination to ensue. It is likely that RNA transport granules are highly dynamic and potentially change their composition during intracellular transport so that a critical function of individual molecules is restricted to individual parts of the localization event. Future investigations will help to improve our understanding of the regulation and composition of heterogeneous granules which will have important implications for the myelination procedure.

## Conflict of Interest Statement

The authors declare that the research was conducted in the absence of any commercial or financial relationships that could be construed as a potential conflict of interest.
